# Improved Cleft Palate Operation

**Published:** 1894-06

**Authors:** Davies Colley

**Affiliations:** London


### Improved Cleft Palate Operation.

BY MR. DAVIES-COLLEY, LONDON.

An improved method for operating for the cure of cleft of the hard and soft palate. The operation may be divided into three stages:

1. An incision is made down to the hard palate in front, and behind through the soft palate, with its centre just internal to the last molar. With a raspatory the muco-periosteum is separated by first incision inward. An incision is made about a quarter of an inch from the cleft in front. It runs parallel to the cleft backward, and is continued to the tip of the uvula, splitting the soft palate to the depth of about three-eighths of an inch in front, and a less amount behind. The muco-periosteum between this incision and the cleft of the hard palate is separated inward with the raspatory as far as the edge of the bone. A

triangular flap is raised from the other side of the palate, in such a way that the anterior extremity is free, and the inner margin runs parallel to the edge of the cleft at a distance of one-sixth of an inch. In the soft palate the incision is continued backward so as to split that structure in the same way as on the other side. The muco-periosteum of the hard palate is separated inward and left attached to the edge of the bone.

2. The mesial flaps are united by fine silk or catgut sutures; and continuously with this union the upper planes of the split soft palate are brought together. A bridge is thus formed across the whole cleft with a mucous surface directed upward, and a raw surface downward.

3. The edge of the triangular flap in front, and of the lower plane of the soft palate behind, is united by silver wires and one or two silk sutures to the edge of the hard and soft palate of the other side. A second bridge is thus formed across the whole cleft, with a raw surface looking upward and a mucous surface downward.

The after-treatment is that of the ordinary operation, except that as there is no tension in the hard palate, and very little in the soft, the sutures may be left in from three to six weeks.

The advantages claimed for this operation are:

1. No tissue has to be pared away.

2. A much larger extent of raw surface is brought into close contact than in the ordinary operation.

3. The tension is small.

4. The upward pressure of the tongue is beneficial, as it presses the lower against the upper bridge.

5. Some advantage is gained by using the muco-periosteum of one side in *front* of the cleft to help in bridging the gap of the hard palate.

The only drawback to the operation is that the application of so many sutures makes it rather longer than the ordinary operation.

In each of the six cases upon which Mr. Davies-Colley has employed this method, union has been complete over at least four-fifths of the cleft.—*Cincinnati Lancet-Clinic.*

